# Baicalin promoted site‐2 protease and not site‐1 protease in endoplasmic reticulum stress‐induced apoptosis of human hepatocellular carcinoma cells

**DOI:** 10.1002/2211-5463.12130

**Published:** 2016-10-24

**Authors:** Zhe Yu, Xin Luo, Chen Wang, Jianhong Ye, Shourong Liu, Lei Xie, Fei Wang, Jianfeng Bao

**Affiliations:** ^1^ Department of Hepatic DiseasesHangzhou Xixi HospitalChina

**Keywords:** apoptosis, baicalin, endoplasmic reticulum stress, hepatocellular carcinoma, site‐2 protease

## Abstract

Baicalin (5,6‐dihydroxy‐7‐*o*‐glucuronide flavone) is an extract from the roots of Chinese herb Huang Qin (*Scutellaria baicalensis* Georgi) and is reported to have antioxidative, antiproliferative, anti‐inflammatory, and anticancer activities. This study aimed to investigate the inhibitory effect of baicalin on human hepatocellular carcinoma (HCC) cells and the involvement of endoplasmic reticulum stress‐induced cell apoptosis. Two human HCC cell lines, HepG2 and SMMC7221, were used in this study. The cells were incubated with baicalin solutions at various concentrations. A 3‐(4,5‐Dimethylthiazol‐2‐yl)‐2,5‐diphenyltetrazolium bromide (MTT) assay was used to assess cell proliferation inhibition; a TUNEL assay was used to evaluate cell apoptosis; small RNA interference was applied to silence IRE1, ATF6, and protein kinase R‐like ER kinase (PERK), which are transmembrane proteins inducing cell apoptosis, and two proteases (S1P and S2P) which cleave ATF6. Real‐time PCR was used to evaluate the silencing effects of specific siRNA. Expression levels of specific proteins were analyzed by western blotting. Baicalin was found to inhibit the proliferation of HCC cells by inducing apoptosis in a concentration‐dependent manner. Elevated expression levels of GRP78, CHOP, p50‐ATF6, and caspase12 were found after baicalin incubation. Compared with IRE1 and PERK silencing, ATF6 knockdown dramatically impaired baicalin's apoptosis‐inducing activity. Furthermore, S2P silencing, rather than S1P silencing, was also found to impair baicalin‐induced HCC cell apoptosis significantly. In conclusion, (a) baicalin inhibits human HCC cells by inducing apoptosis; (b) baicalin induces cell apoptosis by activating ATF6 signaling pathway in endoplasmic reticulum (ER) stress; (c) S2P, rather than S1P is the molecular target for baicalin in inducing ER stress‐mediated HCC cell apoptosis.

**Abbreviations**
ATF6: activating transcription factor 6
ER: endoplasmic reticulum
HCC: hepatocellular carcinoma
IRE1: inositol‐requiring enzyme1
PERK: protein kinase R‐like ER kinase
S1P: site‐1 protease
S2P: site‐2 protease

Hepatocellular carcinoma (HCC) is one of the most common malignant tumors of the digestive system. It is reported that approximately 700 000 deaths are caused annually by HCC which is the third leading cause of cancer‐related deaths worldwide 1. The prognosis of HCC is poor due to the high malignancy. The 5‐year survival rate of HCC is as low as 9% 2. Regular treatments including surgery, chemotherapy, radiotherapy, and liver transplantation could only offer limited clinical outcomes. Some novel approaches, such as anticancer agents extracted from natural herbs 3, attracted oncologists' attention in recent years.

Potent anticancer effects with relatively few side effects characterize the natural anticancer products 4. These agents are considered as potential alternative remedies for curing malignant tumors. Baicalin, also known as 5,6‐dihydroxy‐7‐*o*‐glucuronide flavone, is one of the extracts from roots of Chinese herb Huang Qin (*Scutellaria baicalensis* Georgi) 5. The biological activities of baicalin are various, including antioxidation, antiproliferation, anti‐inflammation, and anticancer activities 6, 7. It was believed that baicalin showed significant anticancer effects on many human cancers including ovarian cancer, lung cancer, pancreatic cancer, and liver cancer 8, 9. However, the specific molecular mechanisms are still vague.

Triggered by several endogenous and exogenous factors such as physiological condition alterations and drugs, the endoplasmic reticulum (ER) stress is initiated. Three transmembrane proteins on the ER membrane are considered as ER sensors, namely inositol‐requiring enzyme1 (IRE1), protein kinase R‐like ER kinase (PERK), and activating transcription factor 6 (ATF6) 10. After ER stress is initiated, the full length of ATF6 is transferred to Golgi complex, where ATF6 (p90) is cleaved by Site‐1 protease (S1P) and Site‐2 protease (S2P) 11, 12. The cleaved segment of ATF6 (p50) then translocates into nucleus to initiate apoptosis‐related gene transcription to induce cell death 13.

Several previous studies revealed that cancer cell death was associated with the anti‐cancer effects of baicalin 9, 14. But the signaling transductions are still unclear. In this study, we investigated the role of ER sensors in baicalin‐mediated ER stress‐induced cell apoptosis of human HCC cells. Moreover, we took a further look into the possible molecular mechanisms and assumed S2P as the potential therapeutic target for HCC. We believe that the result from the current study would not only improve our knowledge about the pharmacological mechanisms of baicalin in inhibiting HCC but also providing new clues for application of baicalin as an alternative medicine in HCC treatment.

## Materials and methods

### Cell culture and treatments

Human HCC cell line HepG2 and SMMC7721 cells were purchased from the American Type Culture Collection (ATCC, Manassas, VA, USA). The cells were maintained in RPMI1640 medium (Gibco, Grand Island, NY, USA) supplemented with 10% FBS (Gibco), l‐glutamine (2.5 mmol·L^−1^; Invitrogen, Carlsbad, CA, USA), penicillin (100 U·mL^−1^; Invitrogen), and streptomycin (100 μg·mL^−1^; Invitrogen). The cells were cultured in an incubator (Thermo, Waltham, MA, USA) providing humidified fresh air (5% CO_2_) at 37 °C. Cultured HepG2 and SMMC7721 cells or cells transfected with small interfering RNA (siRNA) were treated with baicalin solution at various concentrations (0, 20, 40, 60, 80, and 100 μmol·L^−1^) for 48 h.

### Small interfering RNA transfection

The siRNA in this study was used to silence the expressions of ire1, perk, atf6, s1p, and s2p, respectively. The siRNA were designed and synthesized by GenePharma (Shanghai, China). siRNA sequence against ire1 was: 5′‐CAGCACGGACGTCCAGTTTGA‐3′; siRNA sequence against perk was: 5′‐CACAAACTGTATAACCGTTA‐3′; siRNA sequence against atf6 was: 5′‐CAGCAACCAATTTATCAGTTTA‐3′; siRNA sequence against s1p was: 5′‐CAGCCAGCAAUAUCAUUAUUU‐3′; siRNA sequence against s2p was: 5′‐AACAUAGUACCGAUCAGTGTCAUU‐3′. The scrambled siRNA control (Santa Cruz, Santa Cruz, CA, USA) was used as positive control. By using HiPerFect siRNA transfection reagent (Qiagen, Valencia, CA, USA), equal amount of gene‐specific or scrambled siRNA were transfected to human HCC cells according to manufacturer's instructions. After 24‐h culturing, the cells were used for subsequent experiments.

### Cell proliferation assessment

The MTT assay was used to assess the cell proliferation of both HepG2 and SMMC7721 cells in this study. Cultured cells were seeded in culturing plates at density of 6 × 10^3^/well. Then, cells were subjected to baicalin solution at various concentrations for 48 h. Then, 20 μL MTT was added to each well for 4‐h incubation. After that, 100 μL of DMSO was added to each well. The absorbance at 490 nm was measured and recorded by a plate reader (Bio‐Rad, Berkeley, CA, USA). The cell proliferation inhibition rate was calculated by comparing the measured values to that of DMSO‐treated control cells.

### Cell apoptosis evaluation

Terminal dexynucleotidyl transferase (TdT)‐mediated dUTP nick end labeling (TUNEL) assay was used to evaluate the cell apoptosis. After fixed by 2% paraformaldehyde at room temperature for 30 min, the cells were permeablized by 0.1% Triton X100 solution (Solarbio, Beijing, China) for 30 min at room temperature. TUNEL assay kit (Roche, Basel, Switzerland) was used to tag the apoptotic cells according to the manufacturer's instructions. TUNEL‐positive cells were observed by a fluorescence microscope (Olympus, Tokyo, Japan).

### Real‐time PCR

Real‐time PCR was performed to evaluate the silencing effects of siRNA on target genes. Total RNA was extracted from cultured cells by Rneasy MiNi kit (Qiagen) per manufacturer's instructions. Nanodrop spectrophotometer was used to quantify the extracted RNA. After transcribed to cDNA by PrimeScript RT Reagent kit (TaKaRa, Dalian, China), the real‐time PCR was carried out by using SYBR Premix Ex Taq II kit (TaKaRa). Sequences of oligonucleotide primers for perk, ire1, atf6, s1p, and s2p were listed in Table 1. Relative gene quantification was calculated with a comparative threshold cycle method.

**Table 1:** Primers for real‐time PCR

Gene primer	Sequence
ire1	
Forward	5′‐TGGCAAGGGAGTTGAGCTATGGAA‐3′
Reverse	5′‐TGTGCTTTCAAGTGCAGCCAAGAG‐3′
atf6	
Forward	5′‐GATGGAAAAGCCTGCGCA‐3′
Reverse	5′‐GATGGAAAAGCCTGCGCA‐3′
perk	
Forward	5′‐TCCTGCTTTGCATCGTAGCC‐3′
Reverse	5′‐GATGGAAAAGCCTGCGCA‐3′
s1p	
Forward	5′‐ATACAGTTGTGGAATATGGAATAATTG‐3′
Reverse	5′‐ATGTTTCGAGGTATAATTCTCCAATTG‐3′
s2p	
Forward	5′‐GTTGGGGTGCTCATCACTGAA‐3′
Reverse	5′‐CATTACCGTGCTGTAACCATCCAG‐3′
β‐Actin	
Forward	5′‐CTAACAATGAGCTGCGTGTGGC‐3′
Reverse	5′‐CAGGTCCAGACGGAGGATGGC‐3′

### Western blotting

HepG2 and SMMC7221 were lysed by RIPA cell lysis buffer (Santa Cruz). Total protein extracted from harvested cells with Protein Extraction kit (Beyotime, Nanjing, China). Then, the concentrations of protein samples were detected by BCA method with a BCA protein assay kit (Pierce, Waltham, MA, USA). Then, the proteins were subjected to SDS/PAGE and then transferred to polyvinylidene fluoride or NC membranes. Antibodies against GRP78, IRE1, ATF6, PERK, S1P, S2P, CHOP, Caspase12, and GAPDH were used to incubate the membranes. Corresponding secondary antibodies were used to incubate the membranes and the final bands were visualized with an ECL system (Bio‐Rad).

### Statistics

The statistical analysis was carried out with software spss (V.12.0, Boston, MA, USA). Student's *t*‐test and one‐way analysis of variance (ANOVA) were used to analyze the significance of differences. A *P* value < 0.05 was considered statistically significant.

## Results

### Baicalin inhibited cell proliferation of both HepG2 and SMMC7721 cells in a concentration‐dependent manner

**Figure 1:**
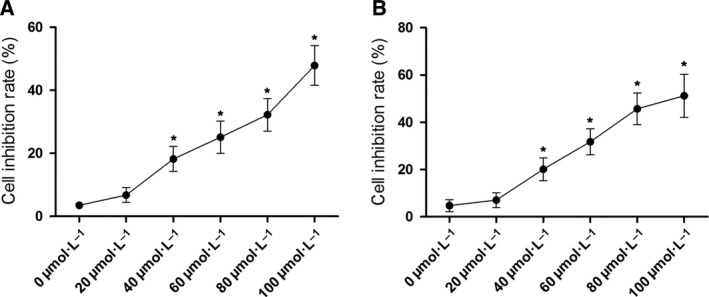
Proliferation inhibitory effect of baicalin on liver cancer cells. Charts demonstrated the results of MTT assay of HepG2 cells (A) and SMMC7721 cells (B) which were incubated with baicalin at incremental concentrations (0, 20, 40, 60, 80, and 100 μmol·L^−1^), respectively. *Differences were statistically significant when compared with previous concentration (*P* < 0.05).

After incubation with serially diluted baicalin solutions (concentrations at 0, 20, 40, 60, 80, and 100 μmol·L^−1^) for 48 h, MTT assay was used to assess cell proliferation in this study. As shown in Fig. 1, baicalin began to show significant inhibitory effect on cell proliferation on HepG2 and SMMC7721 cells at 40 μmol·L^−1^. Thus, 40 μmol·L^−1^ was indicated as the lowest inhibitory concentration (IC). Furthermore, the cell proliferation of HepG2 and SMMC7721 cells were inhibited by baicalin in a concentration‐dependent manner.

### Baicalin induced cell apoptosis of both HepG2 and SMMC7721 cells in a concentration‐dependent manner

**Figure 2:**
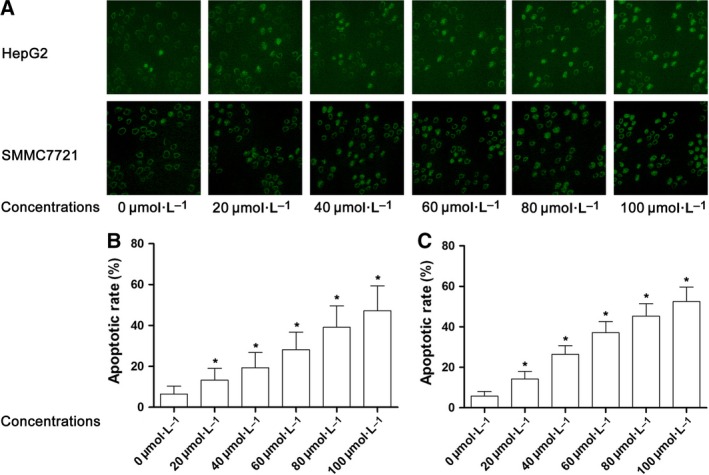
Apoptosis inducing effect of baicalin on liver cancer cells. (A) Captured images of TUNEL assay were demonstrated. HepG2 and SMMC7721 cells were incubated with baicalin at incremental concentrations. Stained cells were TUNEL‐positive. (B) Columns indicated the apoptosis percentage of HepG2 cells after incubation with baicalin at incremental concentrations. (C) Columns indicated the apoptosis percentage of SMMC7721 cells after incubation with baicalin at incremental concentrations. *Differences were statistically significant when compared with previous concentration (*P* < 0.05).

Baicalin solution at 0, 20, 40, 60, 80, and 100 μmol·L^−1^ were used to incubate HepG2 and SMMC7721 cells for 48 h. TUNEL assay was utilized to identify the apoptotic cells. As demonstrated in Fig. 2, baicalin significantly induced apoptosis of both HepG2 and SMMC7721 cells in a concentration‐dependent manner. As baicalin at 80 μmol·L^−1^ showed potent antiproliferative and apoptosis‐inducing effects, this concentration was selected for subsequent mechanism investigations.

### Baicalin promoted ER stress mainly through ATF6 pathway to induce apoptosis in both HepG2 and SMMC7721 cells

**Figure 3:**
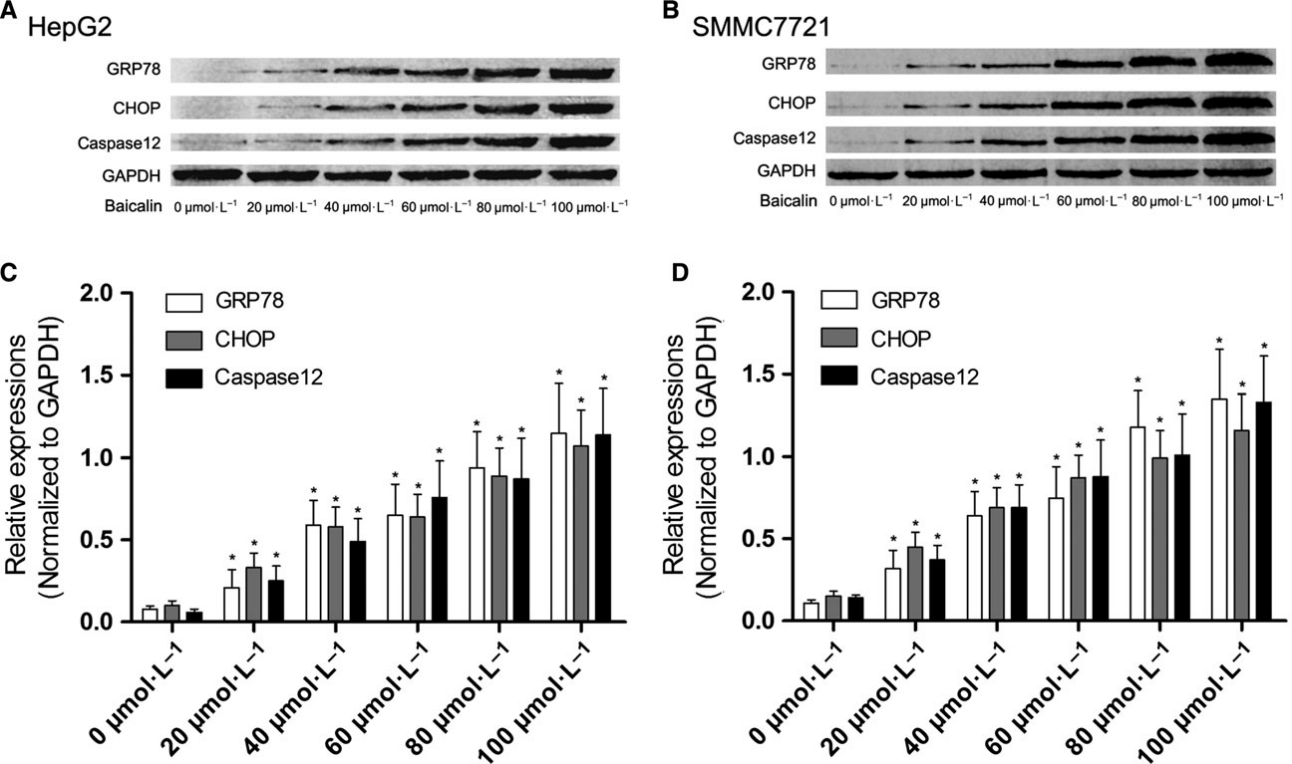
Activation of ERS‐mediated apoptosis signaling transduction in baicalin‐treated liver cancer cells. (A) The immunoblots of GRP78, CHOP and caspase12 in HepG2 cells incubated with baicalin at incremental concentrations. (C) showed the relative expression levels of GRP78, CHOP and caspase12 (GAPDH were used as the internal reference) in HepG2 cells incubated with baicalin at incremental concentrations. (B) The immunoblots of GRP78, CHOP and caspase12 in SMMC7721 cells incubated with baicalin at incremental concentrations. (D) The relative expression levels of GRP78, CHOP, and caspase12 (GAPDH were used as the internal reference) in SMMC7721 cells incubated with baicalin at incremental concentrations. *Differences were statistically significant when compared with previous concentration (*P* < 0.05).

**Figure 4:**
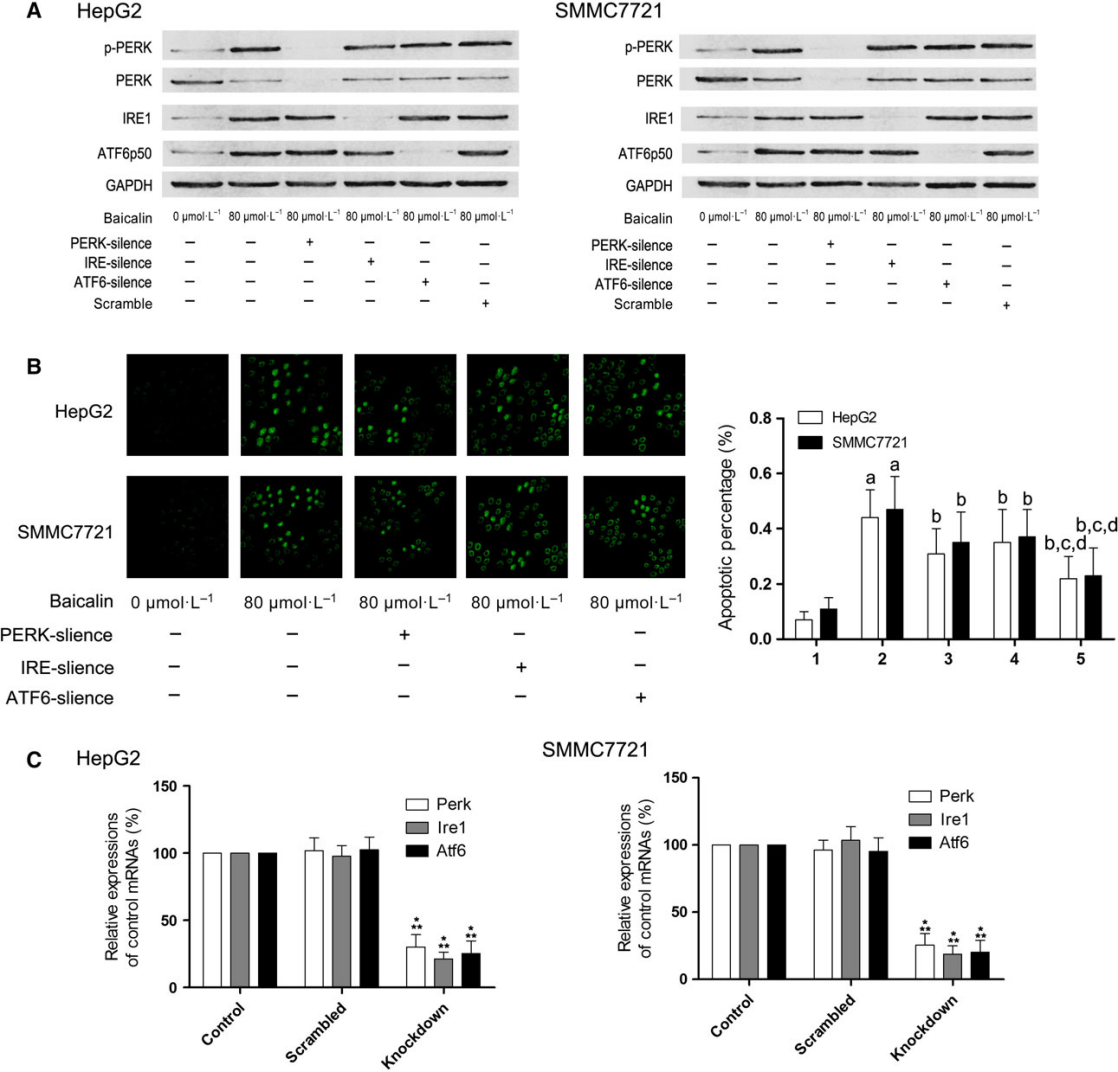
Silencing of PERK, IRE1, ATF6 and its effects on apoptosis in baicalin‐treated liver cancer cells. (A) The immunoblots of p‐PERK, PERK, IRE1, ATF6p50, and GAPDH of HepG2 (left panel) and SMMC7721 (right panel) were demonstrated. (B) The left side of this panel showed the captured images of TUNEL assay in HepG2 and SMMC7721 cells received different treatments. Columns on the right side of this panel indicated the apoptosis percentage of HepG2 and SMMC7721 cells received different treatments. Labels on the bottom of columns: ‘1’ indicated untreated control cells; ‘2’ indicated cells treated with baicalin at 80 μmol·L^−1^; ‘3’ indicated PERK‐silenced cells treated with baicalin at 80 μmol·L^−1^; ‘4’ indicated IRE1‐silenced cells treated with baicalin at 80 μmol·L^−1^; ‘5’ indicated ATF6‐silenced cells treated with baicalin at 80 μmol·L^−1^. Differences were statistically significant (*P* < 0.05) when compared with ^a^ ‘1’ ^b^ ‘2’ ^c^ ‘3’; ^d^ ‘4’. (C) Results of real‐time PCR were shown in this figure. Columns on the left side indicated the relative expression levels of mRNA of perk, ire1, and atf6 in HepG2 cells while columns on the right side indicated the relative expression levels of correspondent mRNA in SMMC7721 cells treated as control, treated with scrambled siRNA, and treated with specific siRNA against target genes, respectively. *Differences were statistically significant when compared with Control (*P* < 0.05); **differences were significant when compared with Scrambled (*P* < 0.05).

HepG2 and SMMC7721 were incubated with baicalin solution at incremental concentrations for 48 h. As a result, as demonstrated in Fig. 3, expression levels of GRP78, CHOP, and caspase12 increased significantly. The silencing effects of siRNA on targeted genes were shown in Fig. 4. mRNA of targeted genes, including perk, ire1, atf6, s1p, and s2p were effectively reduced by specific siRNA. The expression of three ER stress sensor proteins, namely IRE1, PERK, and ATF6 were silenced by specific siRNA, respectively (Fig. 4). The siRNA transfected HepG2 or SMMC7721 cells were then incubated with baicalin at 80 μmol·L^−1^ (Fig. 4). The silence of ATF6 dramatically impaired apoptosis‐ inducing effect of baicalin rather than IRE1 and PERK knockdown (Fig. 4).

### S2P, rather than S1P, was promoted by baicalin which intensified the cleavage of ATF6 and subsequent cell apoptosis

**Figure 5:**
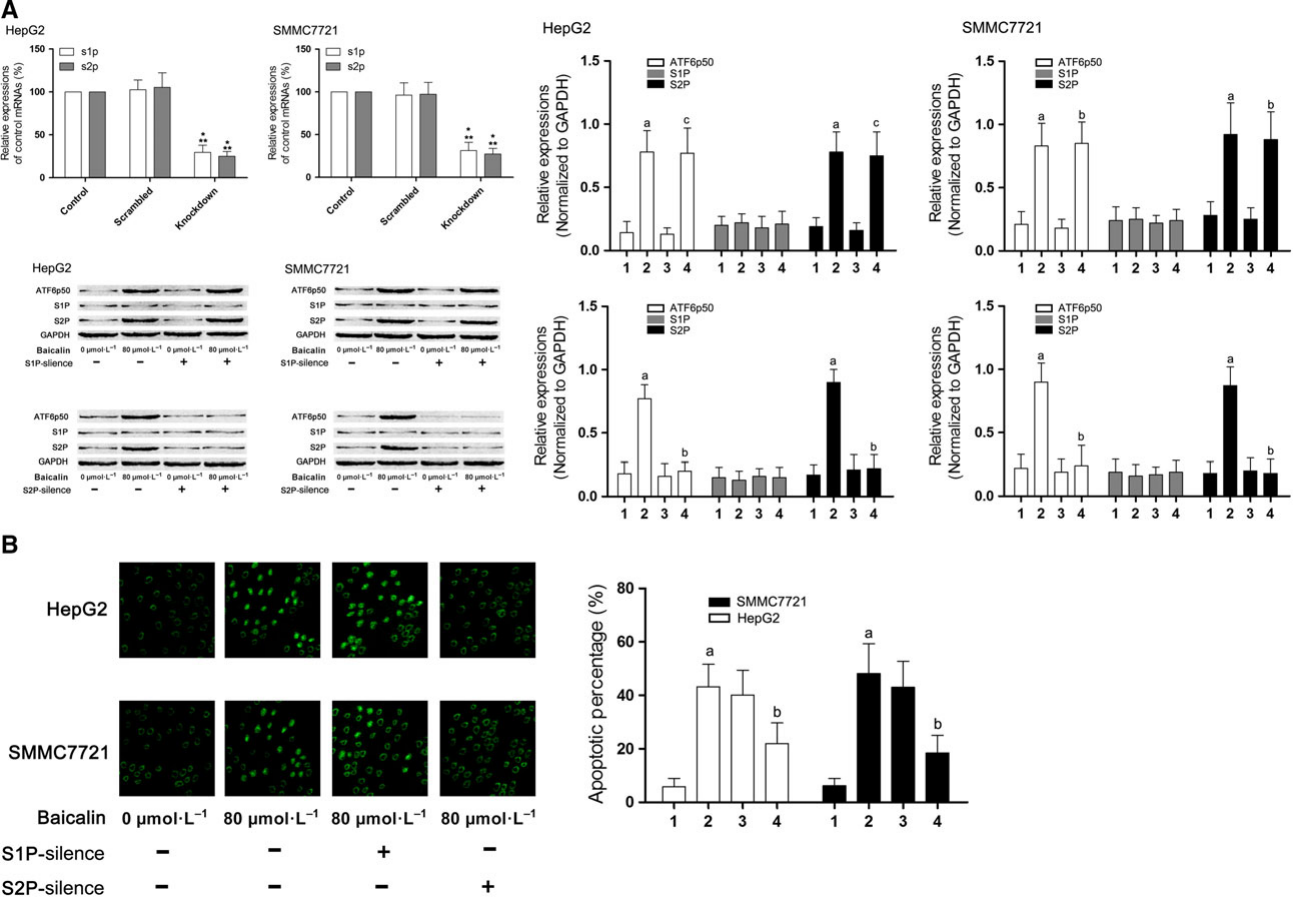
S1P and S2P signaling activation in baicalin‐incubated liver cancer cells effects of their silencing on cell apoptosis. (A) Columns on the upper part of the left side of this figure indicated the relative expression levels of s1p and s2p in HepG2 and SMMC7721 cells treated with specific siRNA against target genes, respectively. *Differences were statistically significant when compared with Control (*P* < 0.05); **differences were significant when compared with Scrambled (*P* < 0.05)]. The immunoblots of ATF6p50, S1P, S2P, and GAPDH of HepG2 (left side) and SMMC7721 (right side) were demonstrated on the lower part of the left panel. Columns on the right panel indicated the relative expression levels of ATF6p50, S1P, and S2P in HepG2 (left side) and SMMC7721 (right side) received different treatments. Labels on the bottom of columns: ‘1’ indicated untreated control cells; ‘2’ indicated cells treated with baicalin at 80 μmol·L^−1^; ‘3’ indicated S1P‐ or S2P‐silenced cells untreated with baicalin; ‘4’ indicated S1P‐ or S2P‐silenced cells treated with baicalin at 80 μmol·L^−1^. (B) The left side demonstrated the captured images of TUNEL assay in HepG2 and SMMC7721 cells received different treatments. Columns on the right side indicated the apoptosis percentages of HepG2 and SMMC7721 cells received different treatments. Labels on the bottom of columns: ‘1’ indicated untreated control cells; ‘2’ indicated cells treated with baicalin at 80 μmol·L^−1^; ‘3’ indicated S1P‐silenced cells treated with baicalin at 80 μmol·L^−1^; ‘4’ indicated S2P‐silenced cells treated with baicalin at 80 μmol·L^−1^. Differences were statistically significant (*P* < 0.05) when compared with ^a^ ‘1’; ^b^ ‘2’; ^c^ ‘3’.

After incubation with baicalin at 80 μmol·L^−1^, the expression level of S2P, rather than S1P, was significantly elevated in both HepG2 and SMMC7721 cells. The expression level of ATF6 cleavage, ATF6p50 increased significantly. siRNA were used to silence the expression of S1P and S2P in HepG2 and SMMC7721 cells, respectively, which were then incubated with baicalin at 80 μmol·L^−1^. As a result, the ATF6p50 expression level decreased significantly in S2P‐silenced cells. Correspondingly, S2P silencing dramatically suppressed the baicalin‐induced apoptosis (Fig. 5).

## Discussion

In this study, two human HCC cell lines, HepG2 and SMMC7721, were used to investigate the anticancer effect of baicalin. We reported that baicalin exerted its anticancer effect by suppressing cell growth through inducing cell apoptosis. Evidenced by increased expression level of ER stress molecular marker GRP78, the ER stress was proved aroused after HCC cells received baicalin incubation. It was assumed in this study that baicalin induced apoptosis of HCC cells through intensified ER stress. We further investigated the molecular mechanisms by using small RNA interfering technique. Three ER stress sensors, namely IRE1, ATF6, and PERK were silenced. Compared with IRE1 and PERK knockdown, ATF6 silencing significantly impaired the apoptosis‐inducing effect of baicalin, implying the death signal in ER stress was transduced by ATF6 pathway. Moreover, expression of the ATF6 splicer, S2P, was found increased dramatically after baicalin incubation. Moreover, the S2P silencing by siRNA also impaired baicalin's apoptosis‐inducing effects on HCC cells, indicating S2P might be the molecular target for baicalin.

Baicalin is a flavonoid extracted from roots of a traditional Chinese herb named *Scutellaria baicalensis* Georgi 15. It has been known that the anticancer spectrum of baicalin is wide 16. The anticancer activities of baicalin against human Burkitt lymphoma 17, lung cancer 18, cervical cancer 19, colon cancer 8, and other human carcinomas were reported. It was indicated that the mechanism of inhibitory effect of baicalin on cancer cells was associated with its apoptosis‐inducing activity 20, 21. In the present study, after incubation with baicalin, the proliferation of HCC cells was dramatically inhibited in a concentration‐dependent manner. Correspondingly, baicalin was found to induce the apoptosis of HCC cells also in a concentration‐dependent manner.

It is generally accepted that cell apoptosis is one of the fundamental physiological and pathological phenomena in mammalian cells. There are mainly three pathways mediating the apoptotic pathway: the mitochondrial pathway (intrinsic pathway), the surface death receptor pathway (extrinsic pathway), and the ER stress‐mediated pathway 22. There are three trans‐membrane proteins located on ER membrane: IRE1, ATF6, and PERK which are considered as the sensors of ER stress. When ER stress is initiated by accumulated abnormal proteins in ER lumen, IRE1, ATF6, and PERK signaling pathways are activated to induce apoptosis 23. In this study, the expressions of IRE1, ATF6, and PERK in human HCC cells were silenced by specific siRNA, respectively, and then the cells were subjected to baicaline incubation. The silencing of IRE1 and PERK failed to impair the baicalin‐induced apoptosis. However, the ATF6 knockdown dramatically reduced baicalin‐induced apoptosis. These results indicated that baicalin induced apoptosis of HCC cells via intensifying the activity of ATF6 signaling pathway in ER stress.

During ER stress, ATF6 is released from the ER membrane and further processed within Golgi complex 24. Golgi complex‐resident S1P and S2P are responsible for processing ATF6 by cleavage 12, 25. After this proteasome‐dependent degradation process, the full‐length ATF6 (p90) is cleaved into the active form p50‐ATF6 which then translocates to nucleus to initiate down‐stream genes encoding apoptotic proteins such as CHOP and caspase12 26. In this study, the after incubation with baicalin, the expression of p50‐ATF6 elevated significantly. This result suggested that the enhanced ATF6 cleavage was one of the critical mechanisms of apoptosis‐inducing activity of baicalin. More specifically, we investigated the involvement of the two proteasomes, S1P and S2P. siRNA were used to knockdown the expressions of S1P and S2P, respectively. We found that S2P knockdown, rather than S1P knockdown, dramatically impaired baicalin's apoptosis‐inducing activity against human HCC cells.

According to the results of this study, we could conclude that: (a) baicalin inhibits human HCC cells by inducing apoptosis; (b) baicalin induces cell apoptosis by activating ATF6 signaling pathway in ER stress; (c) S2P, rather than S1P is the molecular target for baicalin in inducing ER stress‐mediated HCC cell apoptosis.

## Author contributions

ZY wrote the manuscript; CW, JY, SL, and JB carried out the experiments; LX collected the results and participated in the statistics; FW participated in the statistics and revised the manuscript; XL conducted the study and participated in experimental procedures.
